# Accelerating Progress on Ticks and Tick-Borne Diseases in Southeast Asia: Regional Challenges, Evidence Gaps, and Priorities (2023–2025)

**DOI:** 10.3390/pathogens15050511

**Published:** 2026-05-11

**Authors:** Benoit Malleret, Mackenzie L. Kwak, Jean-Marc Chavatte

**Affiliations:** 1 Immunology Translational Research Programme, Department of Microbiology and Immunology, Yong Loo Lin School of Medicine, National University of Singapore, Singapore 117545, Singapore; 2 Laboratory of Parasitology, Department of Disease Control, Faculty of Veterinary Medicine, Hokkaido University, Sapporo 060-0818, Hokkaido, Japan; 3 National Public Health Laboratory, Communicable Diseases Agency, Singapore 308442, Singapore; jean-marc_chavatte@cda.gov.sg

**Keywords:** tick, tick-borne diseases, zoonoses, Southeast Asia

## Abstract

Southeast Asia (SEA) faces persistent gaps in regional understanding and control of ticks and tick-borne diseases (TBDs) despite recent advances (2023–2025). The second international symposium on ticks and TBDs in SEA (Singapore, August 2025), following the inaugural 2023 meeting in Cambodia, served as a catalyst for regional exchange that informed this perspective. SEA’s ecological and host diversity supports complex tick–host–pathogen networks, yet evidence remains fragmented due to uneven sampling that has largely focused on livestock and peri-urban environments. Key constraints include limited taxonomic resolution driven by outdated or incomplete identification keys, under-sampling of soft ticks (Argasidae), and the absence of harmonized, open-access regional reference resources (including DNA barcodes and MALDI-TOF MS spectral databases). While MALDI-TOF MS, proteomics, AI-assisted identification, and next-generation sequencing/metagenomics are increasingly applied, their broader regional uptake is limited by the absence of harmonized, open-access reference resources (including DNA barcodes and MALDI-TOF MS spectral databases). Broad ecological surveys and integrated animal and human surveillance remain limited, and vector competence studies are constrained by the scarcity of SEA-derived tick colonies and cell lines. Regional data and recent findings (2024–2026) confirm circulation of multiple TBPs (including *Anaplasma*, *Babesia*, *Borrelia*, *Coxiella*, *Ehrlichia*, *Rickettsia*, and *Theileria*) and highlight emerging viral findings, including southward reports of *Bandavirus dabieense*. Human infestations and non-communicable tick bite outcomes (e.g., tick paralysis and alpha-gal syndrome) are recognized but remain under-reported due to low clinical awareness and limited diagnostics. Importantly, the diagnostic chain is further disrupted by missed/insufficient specimen collection at the point of care, and by constrained capacity to identify (especially immature) ticks to species level—limitations compounded by the absence of harmonized, open-access regional reference resources. The symposium identified six priorities: (1) full completion and regional validation of tick identification keys for adults (in progress) and immatures (to be initiated), plus an open-access DNA barcode library anchored by curated, voucher-based collections from all SEA countries; (2) harmonization of molecular and proteomic diagnostic platforms, including expansion of regional MALDI-TOF MS and NGS protocols and reference databases; (3) development of tick colonies and cell lines from locally prevalent species to support vector competence, vaccine, and acaricide testing; (4) expansion of One Health surveillance with enhanced ecological sampling at wildlife–livestock–human interfaces; (5) establishment of open-access, region-wide data platforms for integrated tick, TBP, and ecological metadata sharing; and (6) sustained investment in human resources, training, and policy advocacy to raise research and public health visibility of ticks and TBDs.

## 1. Introduction

Southeast Asia (SEA) supports exceptionally diverse tick communities and complex tick–host–pathogen networks across rainforests, mountainous zones, and peri-urban/agricultural landscapes. However, despite growing interest and recent advances, the evidence base on ticks and tick-borne pathogens (TBPs) remains fragmented due to uneven sampling that has largely focused on livestock and peri-urban environments. Recent systematic reviews and regional syntheses (2024–2026) [1,2] reflect an increasing body of documentation on tick and TBP diversity across SEA, whilst highlighting persistent geographic, host, and methodological biases in sampling. Key constraints include limited taxonomic resolution driven by outdated or incomplete identification keys, under-sampling of soft ticks, and the absence of harmonized, open-access regional reference resources (including DNA barcodes and MALDI-TOF MS spectral databases). While new tools (MALDI-TOF MS, proteomics, AI-assisted identification, and next-generation sequencing/metagenomics) are increasingly applied, broad ecological surveys, integrated animal and human surveillance, and vector competence studies remain limited, in part because SEA-derived tick colonies and cell lines are scarce.

In this Opinion article, we provide an updated expert perspective on major regional challenges and priorities for tick and TBD research in SEA, drawing on advances since 2023 and the discussions and outcomes highlighted during the second Southeast Asia Ticks and Tick-borne Diseases Symposium (Singapore, August 2025). The symposium is used here as a source of shared observations and priorities rather than the subject of the manuscript. We organize the discussion around constraints and opportunities in taxonomy and reference resources (Section 3 and Section 4), surveillance and pathogen detection (Section 5 and Section 7), clinical and diagnostic gaps (Section 8 and Section 9), and capacity-building needs and regional priorities (Section 10 and Section 11).

## 2. Symposium Context and Regional Research Momentum (2023–2025)

Recent work (2023–2025) has highlighted both advances and persistent gaps in our understanding of ticks, tick-borne pathogens (TBPs), and their control in the context of SEA’s diverse ecological landscapes and host communities. Here, we provide an updated perspective on research challenges and priorities for ticks and TBDs in SEA, drawing on the regional evidence base and ongoing research momentum and proposing practical future steps for the scientific and public health communities. The second SEA ticks and TBD symposium (Singapore, August 2025), following the June 2023 Cambodia meeting, provided a useful point of reference for regional exchange [3].

## 3. Ecological Diversity and Uneven Sampling in Southeast Asia

SEA’s unique blend of landscapes, ranging from dense rainforests and mountainous zones to dynamic peri-urban and agricultural landscapes, results in an exceptional diversity of tick species and complex host–vector–pathogen networks [4]. Southeast Asia forms part of the broader Asian-Australasian biogeographic belt, spanning regions well known for their tick species richness, with approximately 97 recorded tick species in continental Southeast Asia alone [4]. However, much of maritime Southeast Asia lacks comprehensive national species checklists, suggesting an underestimation of true species diversity. Recent studies conducted between 2023 and 2026 collectively suggest that current species inventories remain incomplete, with new surveys across Cambodia, Malaysia and Thailand updating tick diversity across livestock, companion animal and wildlife hosts [1,5,6,7]. Although geographically smaller than other major regions, this richness is broadly comparable to that of Europe and North America (between 90–110 species) and may approach the highly diverse faunas reported from Africa, East Asia, South America and Australasia [4,8].

Since the first symposium, there have been notable advances in mapping tick fauna and TBP distributions in several countries [3]. These advances include host diversity studies and habitat stratification studies [6,9] that emphasise the importance of studying the evolution of tick-host associations across contrasting ecological gradients. New records and host associations continue to emerge [5,6,7], but the evidence base remains fragmented, and sampling efforts are uneven, with a predominant focus on livestock and peri-urban environments. Taken together, these findings highlight both the progress and limitations of current surveillance efforts.

## 4. Taxonomy Bottlenecks and the Need for Shared Reference Resources

Taxonomic resolution remains a central challenge. While many national and colonial-era keys are outdated or incomplete, country-level checklists for hard tick (Acari: Ixodidae) and soft tick (Acari: Argasidae) are expanding [4]. However, a fundamental limitation still remains in the form of the absence of a comprehensive, unified region-wide morphological identification guide for Southeast Asian ticks. In sharp contrast, major regions of the world benefit immensely from standardized, widely adopted identification frameworks, including Europe [10], Africa [11], Australia [12], Japan [13], North America [14,15] and South America [16,17]. In contrast, Southeast Asia lacks an equivalent consolidated framework, with tick identification in the region relying on a fragmented combination of legacy taxonomic work [18,19,20,21,22] and recent revisions. This need to assemble and reconcile multiple sources across various national reference collections represents a major bottleneck for accurate species identification, contributing to inconsistencies that ripple across subsequent taxonomic studies.

**Figure 1:**
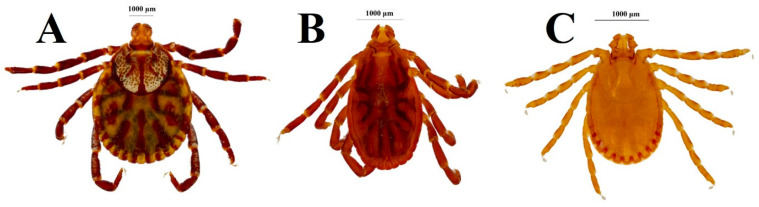
Three major tick genera of medical and economic importance in Southeast Asia; (**A**) *Dermacentor*; (**B**) *Rhipicephalus*, (**C**) *Haemaphysalis* (Images copyright: Kwak, M.L., all rights reserved).

Regional efforts to build integrated morphological and molecular reference resources (including DNA barcoding) have accelerated. However, harmonized, open-access databases are still lacking. The gap is especially pronounced for common genera such as *Dermacentor*, *Rhipicephalus*, and *Haemaphysalis* (Figure 1), where recent morpho-molecular studies have improved species resolution [9,23,24] but also highlight continued dependence on fragmented and non-standardised reference frameworks, which fundamentally constrain accurate identification, comparative ecology, and surveillance across countries [25,26]. Soft ticks, frequently associated with birds and bats in the region, and recognized zoonotic pathogen reservoir in Asia, remain under-sampled, and their associated pathogens are likely underestimated [27].

## 5. Emerging Identification Technologies: MALDI-TOF MS, Proteomics, and AI

The expanding application of MALDI-TOF MS and proteomics for arthropod identification, along with early artificial intelligence (AI)-driven techniques, is transforming tick taxonomy and possibly their surveillance. Nevertheless, the lack of a regional, freely accessible MALDI-TOF MS database similar to GenBank remains a barrier to widespread adoption, especially for resource-limited settings [3]. In practice, current use in the region is therefore best described as expanding but uneven, with progress occurring alongside persistent constraints in shared reference resources and harmonized implementation. Ongoing technical innovation includes the development of homegrown spectral databases, AI-coupled species identification, and non-targeted proteomics for TBP detection.

## 6. Host Diversity, One Health Approaches, and Passive Surveillance Opportunities

SEA’s vertebrate biodiversity is both an asset and a research challenge. Intensive studies on domestic animals, particularly dogs [28] and cattle, are now being complemented by increased efforts in wildlife and peri-domestic environments, with growing interest in companion animals as sentinels for zoonotic TBDs [29,30,31]. The One Health framework is gaining traction, integrating wildlife, livestock, and human health data. Still, the roles of synanthropic and invasive species in maintaining tick populations and facilitating TBPs spillover are only beginning to be understood. Passive surveillance through zoos and conservation organizations is a promising avenue for expanding representative host and tick sampling.

In addition, citizen science programmes represent a valuable complementary surveillance approach that remains underutilised in this region. Community-engaged tick monitoring initiatives such as TICKMAP in New York have successfully generated large spatial datasets supporting early detection of emerging tick-borne disease risks [32], with similar programmes being implemented in Europe, including the UK [33] or Spain [34]. Such programmes can be adapted to Southeast Asia, where formal surveillance capacity remains uneven, leveraging public participation to expand geographic coverage whilst generating valuable public health data [35,36].

## 7. Tick-Borne Pathogen Detection Advances and Remaining Surveillance Gaps

Progress in TBPs detection is significant, with regional data confirming circulation of *Anaplasma*, *Babesia*, *Borrelia*, *Coxiella*, *Ehrlichia*, *Rickettsia*, and *Theileria* [37,38,39]. Veterinary pathogens such as *Babesia vogeli*, *Ehrlichia canis*, and *Theileria orientalis* impact animal health and livestock economies, while zoonotic risks from rickettsiae and borreliae are increasingly documented [40,41,42,43,44]. Nevertheless, most TBP detection relies on convenience sampling and focused clinical studies rather than broad ecological surveys or animal and human surveillance programs. The adoption of next-generation sequencing (NGS) and metagenomic approaches is facilitating the discovery of known and novel viruses, including Langat virus and new orthonairoviruses, but comprehensive vector competence studies in local tick species remain rare [45].

## 8. Viral Tick-Borne Diseases and Constraints on Vector Competence Research

Viral tick-borne diseases are among the least characterized, but the region is seeing notable advances in detection methodologies and early virus isolation efforts. During the symposium, the expansion of the *Bandavirus dabieense*, the agent causing the Severe Fever with Thrombocytopenia Syndrome, was confirmed southward in SEA with reports from Cambodia, Thailand and East Malaysia [46,47]. These reports highlight a potential emerging risk for the region and reinforce the need for expanded surveillance and virus detection efforts, including the increasing use of next-generation sequencing (NGS) and metagenomic approaches described in this manuscript. However, the evidence summarized here does not allow comparison of transmission efficiency between SEA and East Asia, nor does it provide data on genetic variation linked to increased pathogenicity—both of which remain key open questions. The lack of laboratory tick colonies and tick cell lines derived from SEA tick species remains a limiting factor for vector competence research, functional studies, and acaricide testing [3,48].

## 9. Human Tick Infestations, Diagnostic Constraints, and Clinical Awareness

Within the region, the genera *Dermacentor* and *Haemaphysalis* are species rich, abundant, and contribute to large numbers of human infestations [49]. However, building meaningful knowledge on human-tick interactions continues to be hampered by a key breakpoint at first clinical contact: a lack of awareness of the need for sample collection by clinicians dealing with tick infestations. Additionally, efforts are hampered by limited diagnostic ability, representing a second break point in the chain (identification/confirmation), especially given that most infestations are caused by immature ticks, which can be challenging to identify to species level by non-taxonomists. These constraints align with broader regional limitations described elsewhere in this manuscript, including outdated or incomplete identification keys and the absence of harmonized, open-access reference resources (DNA barcodes and MALDI-TOF MS spectral databases), which together restrict accurate identification and surveillance across countries.

## 10. Non-Communicable Tick Bite Outcomes as Emerging Clinical Concerns

Non-communicable outcomes of tick bites (including tick paralysis and allergic syndromes such as alpha-gal syndrome) are recognized as emerging clinical concerns in the region. However, under-reporting due to low clinical awareness and limited acarological diagnostics persists. In practice, the critical break points include (i) failure to collect and retain tick specimens during clinical encounters (as noted in Section 8), and (ii) limited capacity to perform acarological identification—particularly for immature ticks—thereby preventing reliable species-level attribution and weakening downstream reporting and surveillance. Greater education and inclusion of tick exposure in clinical workflows are needed, as highlighted during symposium roundtable discussions [50].

## 11. Persistent Capacity Gaps and the Need for Regional Resource Sharing

Despite the technical and conceptual advances, several persistent challenges remain. These include a shortage of trained tick taxonomists and medical/veterinary entomologists, limited research visibility and funding, insufficient capacity-building and laboratory infrastructure, and difficulties in establishing and maintaining laboratory tick colonies for vector competence studies [3]. The symposium emphasized the necessity of strengthening regional networks and resource sharing to address these gaps.

## 12. Six Regional Research Priorities Identified by the Symposium

Moving forward, six research priorities stand out: (1) Full completion and regional validation of a tick identification keys both for the adults (work in progress) and immatures stages (to be initiated) and open-access DNA barcode library, anchored by curated, voucher-based collections from all SEA countries; (2) Harmonization of molecular and proteomic diagnostic platforms, including expansion of regional MALDI-TOF MS and NGS protocols and reference databases; (3) Development of tick colonies and cell lines from locally prevalent species to support laboratory-based research on vector competence, vaccine, and acaricide testing; (4) Expansion of One Health surveillance with enhanced ecological sampling at wildlife–livestock–human interfaces; (5) Establishment of open-access, region-wide data platforms for integrated tick, TBP, and ecological metadata sharing; and (6) Sustained investment in human resources, training, and policy advocacy to raise research and public health visibility of ticks and TBDs.

## 13. Conclusions: From Momentum to Sustainable Surveillance and Intervention

In conclusion, SEA tick and TBD research has entered a new phase since 2023, with progress in technical capacity, regional networking, and conceptual frameworks. However, the challenges of taxonomy, surveillance, laboratory infrastructure, and clinical awareness require continued attention, investment, and cross-sectoral collaboration. The next years must translate collective momentum into sustainable operational surveillance and targeted intervention, ensuring SEA is equipped to address the evolving risks posed by ticks and tick-borne pathogens in a changing world.

## Data Availability

No new data were created or analysed in this study.

## References

[1] Soe B.K., Huertas-Lopez A., Sato H., Adisakwattana P. (2026). Ticks and tick-borne pathogens in Southeast Asia: Meta-analysis. Acta Trop..

[2] Burn L., Fletcher M.A., Montiel J., Ghia C.J., Dantas A., Kelly P.H., Stark J.H. (2025). Lyme borreliosis epidemiology. Vector-Borne Zoonotic Dis..

[3] Yean S., Prasetyo D.B., Marcombe S., Hadi U.K., Kazim A.R., Tiawsirisup S., Chinh V.D., Matsuno K., Low V.L., Bonnet S. (2024). Challenges for ticks and tick-borne diseases research in Southeast Asia: Insight from the first international symposium in Cambodia. PLoS Neglected Trop. Dis..

[4] Petney T.N., Saijuntha W., Boulanger N., Chitimia-Dobler L., Pfeffer M., Eamudomkarn C., Andrews R.H., Ahamad M., Putthasorn N., Muders S.V. (2019). Ticks (Argasidae, Ixodidae) and tick-borne diseases of continental Southeast Asia. Zootaxa.

[5] Yean S., Krib D., Chea R., Sum S., Ren T., San S., Maquart P.-O., Boyer S. (2026). Tick diversity and pathogens in Cambodia. Vet. Parasitol. Reg. Stud. Rep..

[6] Husin N.A., AbdulRahim M.H.S., Halim M.R.A., AbdulHalim A.A., Mohd-Redzuan M.A.A., Azman S.N.A., Ganasen T., Sahimin N., Low V.L., Jiliun E.A. (2026). Tick-host associations in Malaysia. Parasites Vectors.

[7] Narapakdeesakul D., Myint S.Y.P.P., Aung Z.T., Junsiri W., Thanasak J., Pongtheerat T., Beugnet F., Taweethavonsawat P. (2026). Tick-associated microorganisms in Thailand. Curr. Res. Parasitol..

[8] Guglielmone A.A., Robbins R.G., Apanaskevich D.A., Petney T.N., Estrada-Peña A., Horak I.G. (2014). The Hard Ticks of the World.

[9] Lah E.F.C., George E., Abidin S.Z., Ahamad M., A Apanaskevich D., Yaakop S. (2026). Species composition of ticks in Malaysia. J. Med. Entomol..

[10] Estrada-Peña A., Mihalca A.D., Petney T.N. (2017). Ticks of Europe and North Africa.

[11] Walker A.R. (2003). Ticks of Domestic Animals in Africa.

[12] Barker S.C., Walker A.R. (2014). Ticks of Australia. Zootaxa.

[13] Yamaguti N. (1971). Ticks of Japan, Korea, and the Ryukyu Islands.

[14] Keirans J.E., Durden L.A. (1998). Ticks (Acari: Ixodida) of the United States: A species list. J. Med. Entomol..

[15] Keirans J.E., Durden L.A. (1998). Illustrated key to nymphs of the tick genus Amblyomma (Acari: Ixodidae) found in the United States. J. Med. Entomol..

[16] Barros-Battesti D.M., Arzua M., Bechara G.H. (2006). Carrapatos de Importância Médico-Veterinária da Região Neotropical: Um Guia Ilustrado para Identificação de Espécies.

[17] Dantas-Torres F., Martins T.F., Muñoz-Leal S., Onofrio V.C., Barros-Battesti D.M. (2019). Ticks (Ixodida: Argasidae, Ixodidae) of Brazil: Updated species checklist and taxonomic keys. Ticks Tick-Borne Dis..

[18] Hoogstraal H., Kim K.C., Kim K.C. (1985). Tick and mammal coevolution, with emphasis on Haemaphysalis. Coevolution of Parasitic Arthropods and Mammals.

[19] Hoogstraal H., Wassef H.Y. (1973). The Haemaphysalis ticks (Ixodoidea: Ixodidae) of birds. 3. H. (Ornithophysalis) subgen. n.: Definition, species, hosts, and distribution in the Oriental, Palearctic, Malagasy, and Ethiopian faunal regions. J. Parasitol..

[20] Voltzit O.V. (2007). A review of neotropical Amblyomma species (Acari: Ixodidae). Acarina.

[21] Filippova N.A. (1997). Ixodid ticks of the subfamily Amblyomminae. Fauna of Russia and Neighbouring Countries. Arachnida.

[22] Walker J.B. (2000). The Genus Rhipicephalus.

[23] Teo E.J.M., Apanaskevich D.A., Barker S.C., Nakao R. (2024). Dermacentor (Indocentor) auratus Supino 1897: Potential geographic range, and medical and veterinary significance. Acta Trop..

[24] Tan L.P., Hamdan R.H., Hassan B.N.H., Reduan M.F.H., Okene I.A.-A., Loong S.K., Khoo J.J., Samsuddin A.S., Lee S.H. (2021). Rhipicephalus tick review in Southeast Asia. Pathogens.

[25] Kwak M.L. (2018). Ticks in the Lion City: A preliminary review of the tick fauna of Singapore. Exp. Appl. Acarol..

[26] Guglielmone A.A., Nava S., Robbins R.G. (2023). Geographic distribution of the hard ticks (Acari: Ixodida: Ixodidae) of the world by countries and territories. Zootaxa.

[27] Kazim A., Houssaini J., Ehlers J., Tappe D., Heo C. (2021). Soft ticks in the island nations of Southeast Asia. Acta Trop..

[28] Perera V., Eremeeva M.E., Dangolla A., Wijeratne S., Rajakaruna R.S. (2025). Canine anaplasmoses in South and Southeast Asia. Vet. Parasitol. Reg. Stud..

[29] Tanskul P., Stark H.E., Inlao I. (1983). A checklist of ticks of Thailand. J. Med. Entomol..

[30] Jongejan F., Uilenberg G. (2004). The global importance of ticks. Parasitology.

[31] De La Fuente J., Antunes S., Bonnet S., Cabezas-Cruz A., Domingos A.G., Estrada-Peña A., Johnson N., Kocan K.M., Mansfield K.L., Nijhof A.M. (2017). Tick-Pathogen Interactions and Vector Competence. Front. Cell. Infect. Microbiol..

[32] Hart C.E., Bhaskar J.R., Reynolds E., Hermance M., Earl M., Mahoney M., Martinez A., Petzlova I., Esterly A.T., Thangamani S. (2022). Community engaged tick surveillance and tickMAP as a public health tool to track emerging tick-borne diseases in New York. PLoS Glob. Public Health.

[33] Hansford K.M., Brown F.V., Biddlecombe S.M., Yardley J., Luce E.O., Gandy S., Johnston C.J., Jones N., Abbott A.J., Mackenzie B. (2026). Mapping ticks (Acari: Argasidae, Ixodidae) and informing local public action: Insights from the United Kingdom Tick Surveillance Scheme (2021–2024). Ticks Tick-Borne Dis..

[34] Millán J., Rodríguez-Pastor R., Muñoz-Hernández C., Sánchez-Sánchez M., Moraga-Fernández A., Fernández-Ruiz N., Fernández de Mera I.G., Estrada-Peña A. (2026). Bites of Knowledge: Ticks and Tick-Borne Pathogens Unveiled Through a Citizen Science Programme in Northern Spain. Zoonoses Public Health.

[35] Nieto N.C., Porter W.T., Wachara J.C., Lowrey T.J., Martin L., Motyka P.J., Salkeld D.J. (2018). Using citizen science to describe the prevalence and distribution of tick exposure in the United States. PLoS ONE.

[36] Kampen H., Medlock J.M., Vaux A.G.C., Koenraadt C.J.M., van Vliet A.J.H., Bartumeus F., Oltra A., Sousa C.A., Chouin S., Werner D. (2015). Approaches to passive mosquito and tick surveillance in the EU. Parasites Vectors.

[37] Kazim A.R., Ng S.Y., Zaini N.A., Low V.L., Houssaini J., Tappe D., Heo C.C. (2025). Molecular detection of a relapsing fever Borrelia in Dermacentor auratus infesting a human. Trans. R. Soc. Trop. Med. Hyg..

[38] Bock R., Jackson L., De Vos A., Jorgensen W. (2004). Babesiosis of cattle. Parasitology.

[39] Jirapattharasate C., Adjou Moumouni P.F., Cao S., Iguchi A., Liu M., Wang G., Zhou M., Vudriko P., Efstratiou A., Changbunjong T. (2017). Molecular detection and genetic diversity of bovine *Babesia* spp., *Theileria orientalis*, and *Anaplasma marginale* in beef cattle in Thailand. Parasitol. Res..

[40] Colella V., Nguyen V.L., Tan D.Y., Lu N., Fang F., Zhijuan Y., Wang J., Liu X., Chen X., Dong J. (2020). Zoonotic vectorborne pathogens of dogs and cats in Asia. Emerg. Infect. Dis..

[41] Sharifah N., Heo C.C., Ehlers J., Houssaini J., Tappe D. (2020). Ticks and tick-borne pathogens in Southeast Asia. Acta Trop..

[42] Prasetyo D.B., Fiorenzano J.M., Nop D., Noch N., Huot B., Mom S., Prum S., Chhe V., Dul S., Heang V. (2024). Molecular detection of Rickettsia in Cambodia. PLoS Neglected Trop. Dis..

[43] Kelly G.C., Rachmat A., Tran L.K., Supaprom C., Phireak H., Prom S., Sopheab H., Cleary N., von Fricken M., Farris C.M. (2025). Rickettsiosis in Cambodia. Emerg. Infect. Dis..

[44] Yean S., Maquart P.-O., Delvallez G., Boyer S. (2026). First evidence of human borreliosis in Cambodia. Int. J. Infect. Dis..

[45] Xu Y., Wang J. (2024). The Vector Competence of Asian Longhorned Ticks in Langat Virus Transmission. Viruses.

[46] Saba Villarroel P.M., Chaiphongpachara T., Nurtop E., Laojun S., Pangpoo-Nga T., Songhong T., Supungul D., Baronti C., Thirion L., Leaungwutiwong P. (2024). Seroprevalence study in humans and molecular detection in *Rhipicephalus sanguineus* ticks of severe fever with thrombocytopenia syndrome virus in Thailand. Sci. Rep..

[47] Pérez L.J., Baele G., Hong S.L., Cloherty G.A., Berg M.G. (2024). Ecological changes exacerbating the spread of invasive ticks have driven the dispersal of severe fever with thrombocytopenia syndrome virus throughout Southeast Asia. Mol. Biol. Evol..

[48] Bell-Sakyi L., Darby A., Baylis M., Makepeace B.L. (2018). The Tick Cell Biobank: A global resource for in vitro research on ticks and the pathogens they transmit. Ticks Tick-Borne Dis..

[49] Kwak M.L., Chavatte J.-M., Hsu C.-D., Ng A., Lee B.P.Y.-H., Bin Nazir N., Abas N.F.M., Lee E.Q.H., Nakao R., Malleret B. (2025). Nation-wide surveillance of tick infestations in Singapore. Ticks Tick-Borne Dis..

[50] Nuttall P.A. (2023). Tick saliva and its role in pathogen transmission. Wien. Klin. Wochenschr..

